# Supplementary material for: Prevalence and Characteristics of Invasive Staphylococcus argenteus among Patients with Bacteremia in Hong Kong
Source: Microorganisms. 2023 Sep 28;11(10):2435. doi: 10.3390/microorganisms11102435 (PMC10609611; doi:10.3390/microorganisms11102435)
Supplement: Supplementary file 1 [file microorganisms-11-02435-s001.zip › SupplementaryFile.docx]

**Supplementary Table S1** – Primers and probes of the multiplex real-time PCR for *Staphylococcus argenteus* identification used in this study

| **Name** | **Sequence** | **Concentration** | **Reference** |
| --- | --- | --- | --- |
| *S. aureus sau* gene | |  |  |
| sau_F | 5’-GACCTAATTGCTGCAACTGATCG-3’ | 0.3uM | [35] |
| sau_R | ACTCGTGGTGGTCATATGGAAGC | 0.3uM |  |
| sau_Pb | FAM-AAATCTTCAGCATTATGAATAAATTCGTAACG-3IABkFQ | 0.2uM |  |
| *S. aureus nuc* gene |  |  |  |
| nuc263-F | 5’-AAAGCGATTGATGGTGATACGGTT-3’ | 0.3uM | [36] |
| nuc355-R | 5’-TGCTTTGTTTCAGGTGTATCAACCA-3’ | 0.3uM |  |
| nuc294-P | FAM-ATGTACAAA/ZEN/GGTCAACCAATGACATTYAGA-3IABkFQ | 0.2uM |  |
| *mecA* gene |  |  |  |
| mecA_F | 5’- CATTGATCGCAACGTTCAATTT-3’ | 0.3uM | This study |
| mecA_R | 5’- TGGTCTTTCTGCATTCCTGGA-3’ | 0.3uM |  |
| mecA_Pb | HEX-TGGAAGTTAGATTGGGATCATAGCGTCAT-3IABkFQ | 0.2uM |  |
| *S. argenteus* *coa* gene |  |  |  |
| coa_F2 | 5’-AAGGACCAGACTTCCCAACG-3’ | 0.5uM | This study |
| coa_R2 | 5’-AATTTCCGCTTAATGAAGGCG-3’ | 0.5uM |  |
| coa_Pb2 | Cy5-TTACAACAG/TAO/GTGCAGATTCGTTACT-3IAbRQSp | 0.25uM |  |
